# ^TM^REC: A Database of Transcription Factor and MiRNA Regulatory Cascades in Human Diseases

**DOI:** 10.1371/journal.pone.0125222

**Published:** 2015-05-01

**Authors:** Shuyuan Wang, Wei Li, Baofeng Lian, Xinyi Liu, Yan Zhang, Enyu Dai, Xuexin Yu, Fanlin Meng, Wei Jiang, Xia Li

**Affiliations:** 1 College of Bioinformatics Science and Technology, Harbin Medical University, Harbin, 150081, P. R. China; 2 EpiRNA Lab, Institutes of Biomedical Sciences, Fudan University, Shanghai, 200032, P. R. China; 3 School of Life Sciences and Biotechnology, Shanghai Jiao Tong University, Shanghai, 200240, P. R. China; 4 Shanghai Center for Bioinformation Technology, Shanghai, 201203, P. R. China; German Cancer Research Center (DKFZ), GERMANY

## Abstract

Over the past decades, studies have reported that the combinatorial regulation of transcription factors (TFs) and microRNAs (miRNAs) is essential for the appropriate execution of biological events and developmental processes. Dysregulations of these regulators often cause diseases. However, there are no available resources on the regulatory cascades of TFs and miRNAs in the context of human diseases. To fulfill this vacancy, we established the ^TM^REC database in this study. First, we integrated curated transcriptional and post-transcriptional regulations to construct the TF and miRNA regulatory network. Next, we identified all linear paths using the Breadth First Search traversal method. Finally, we used known disease-related genes and miRNAs to measure the strength of association between cascades and diseases. Currently, ^TM^REC consists of 74,248 cascades and 25,194 cascade clusters, involving in 412 TFs, 266 miRNAs and 545 diseases. With the expanding of experimental support regulation data, we will regularly update the database. ^TM^REC aims to help experimental biologists to comprehensively analyse gene expression regulation, to understand the aetiology and to predict novel therapeutic targets.^TM^REC is freely available at http://bioinfo.hrbmu.edu.cn/TMREC/.

## Introduction

TFs and miRNAs are considered two types of main regulators [1]. TFs have been well characterised in most gene transcriptional events, and the interactions between TFs have also been reported in several previous studies [2]. MiRNAs are single-stranded small non-coding RNA molecules, which have been reported as another prominent type of regulators at the posttranscriptional level [3]. Over the past decades, studies have reported that the combinatorial regulation of TFs and miRNAs is essential for the appropriate execution of biological events and developmental processes [4–6]. Dysregulations of these processes often contribute to disease [7]. Compared with the complex topological structures of regulatory networks, cascades have simpler linear regulations, which can be elucidated and experimentally validated [8]. Currently, several studies have experimentally confirmed that regulatory cascades play important roles in many biological processes. For example, Gao *et al* reported a novel cascade to regulate memory and plasticity via *SIRT1* and *miR-134*, in which *SIRT1* regulated the expression of *miR-134*, and *miR-134* subsequently downregulated *CREB* and *BDNF*. To the best of our knowledge, upstream regulators may be crucial for understanding the aetiology and prediction of novel therapeutic targets. The authors suggested that *SIRT1* had its value to be a potential therapeutic target for the treatment of central nervous system disorders [9]. However, there is no database on the regulatory cascades of TFs and miRNAs in human diseases. Thus, construction of the combinatorial TF and miRNA regulatory networks and identification of regulatory cascades in the context of human diseases are invaluable.

## Results

**Fig 1:**
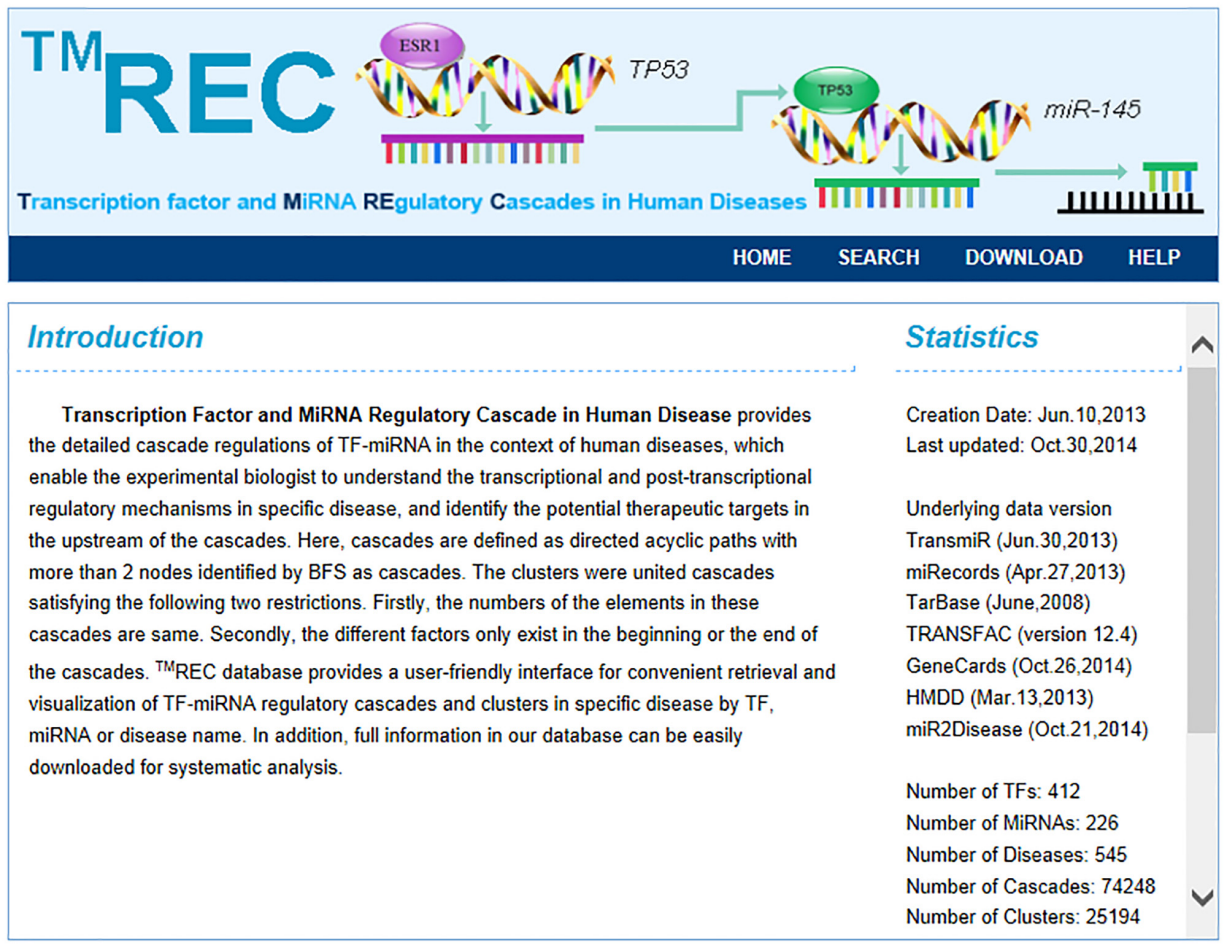
A snapshot of the graphical user interface to the database.

Here, we developed ^TM^REC, a public database for the retrieval and visualisation of TFs and miRNAs regulatory cascades in human diseases. The coordinated regulation of TFs and miRNAs may be involved in various biological processes, and the disruption of this coordination may lead to complex human diseases [10]. Moreover, the regulatory cascade is easily elucidated and validated. From disease-related cascades, we may find key factors located upstream of the pathway and infer the cause of disease onset. We can also predict novel disease related TFs or miRNAs from the regulatory cascades. Thus, identifying regulatory cascades is crucial for dissecting the pathology of complex diseases. The Breadth First Search (BFS) traversal method was used to extract cascades from the TF and miRNA regulatory network. The BFS begins at a root node and inspects all the neighboring nodes. Then for each of those neighbor nodes in turn, it inspects their neighbor nodes which were unvisited. After visiting all the nodes reachable to the root, we can obtain the pathways through backtracking process. In this study, each node in the network was regarded as the root node and a filtration was conducted to get the final results (Details in S1 File). Finally, 74248 cascades, which consisted of 412 TFs and 266 miRNAs were obtained. Furthermore, we identified 25194 clusters according to the rule described in the Methods section. After disease annotation, 545 diseases were integrated into the filtered cascades. All these information was included in ^TM^REC. A snapshot of the graphical user interface to the database is shown in Fig 1. We marked date of last update as well as the version of underlying databases on the statistics page. Currently, ^TM^REC can query single miRNA, single TF, single disease or in combination, and provides cascade searching as well as cluster searching. The final cascades and clusters can be freely downloaded on the download page.

**Fig 2:**
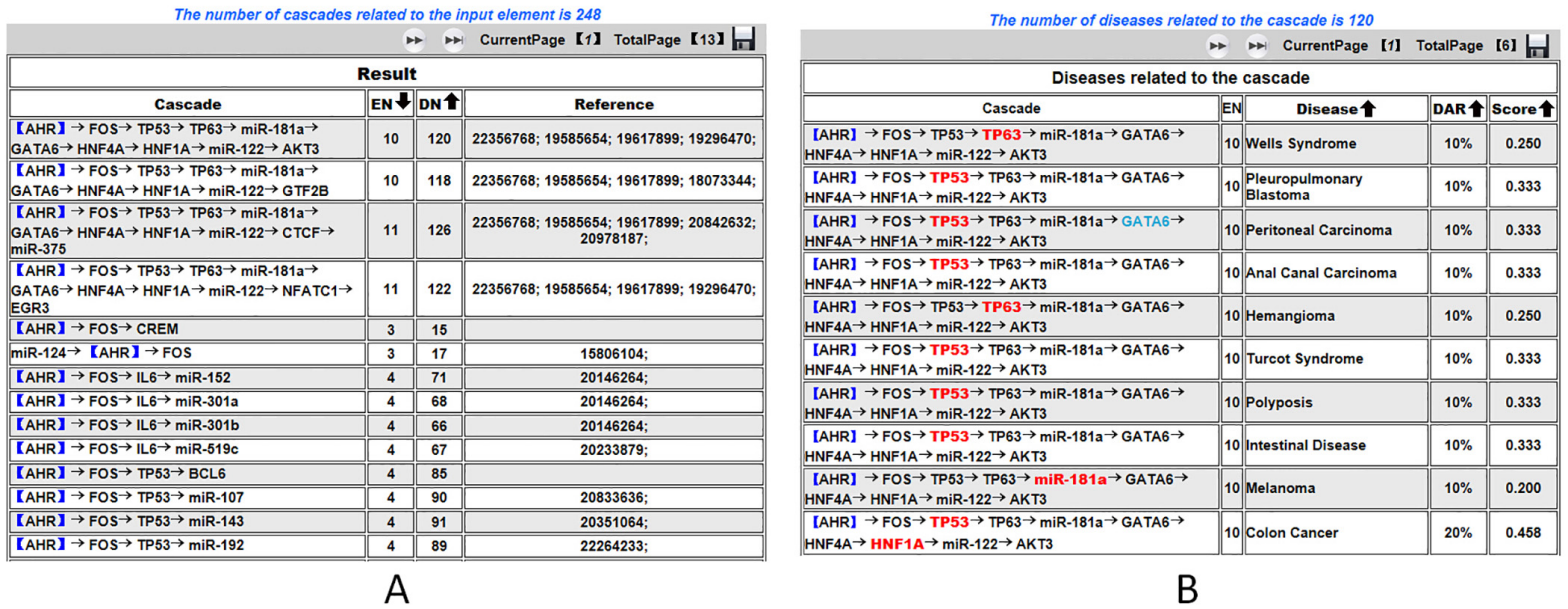
Illustration cascade search result by *AHR*.

In cascade searching, when users select a TF, miRNA or both of TF and miRNA, a sorted cascade list containing the inputted factors, elements number (EN) and annotated diseases number (DN) will be returned. Here, EN represents the number of elements in the listed cascade, and DN is the total number of diseases that are annotated in the cascade. For example, we queried cascades related to *AHR*. First, we selected *AHR* in the TF pull-down list. Next, we clicked the Cascade button to retrieve the results. In the result page, a cascade list containing the inputted factor *AHR*, the corresponding EN and DN was returned (as shown in Fig 2A). Each line represents a cascade, in which the inputted factor *AHR* is included in blue brackets. Each page presents 20 cascades, and all of the results can be browsed using the flip key. Detailed disease information of the cascade will be displayed when the desired cascade is clicked. For example, we click on the cascade marked with the red box in Fig 2A. The result page contains the selected cascade along with the EN, annotated diseases, disease annotated ratio (DAR) and Score (defined in the Methods section), in which disease related TFs and miRNAs are marked in red (as shown in Fig 2B). In addition, users can click the desired element in the cascade to execute an immediate search of another cascade. Furthermore, we support combinatorial searching. Users can input any combination of TF, miRNA or disease simultaneously. If users select a disease as a query, then the result page will return the cascades that contain at least one disease related element, EN, selected disease, the DAR and Score. All of the query results in each step can be downloaded by clicking the save button presented on each result page.

**Fig 3:**
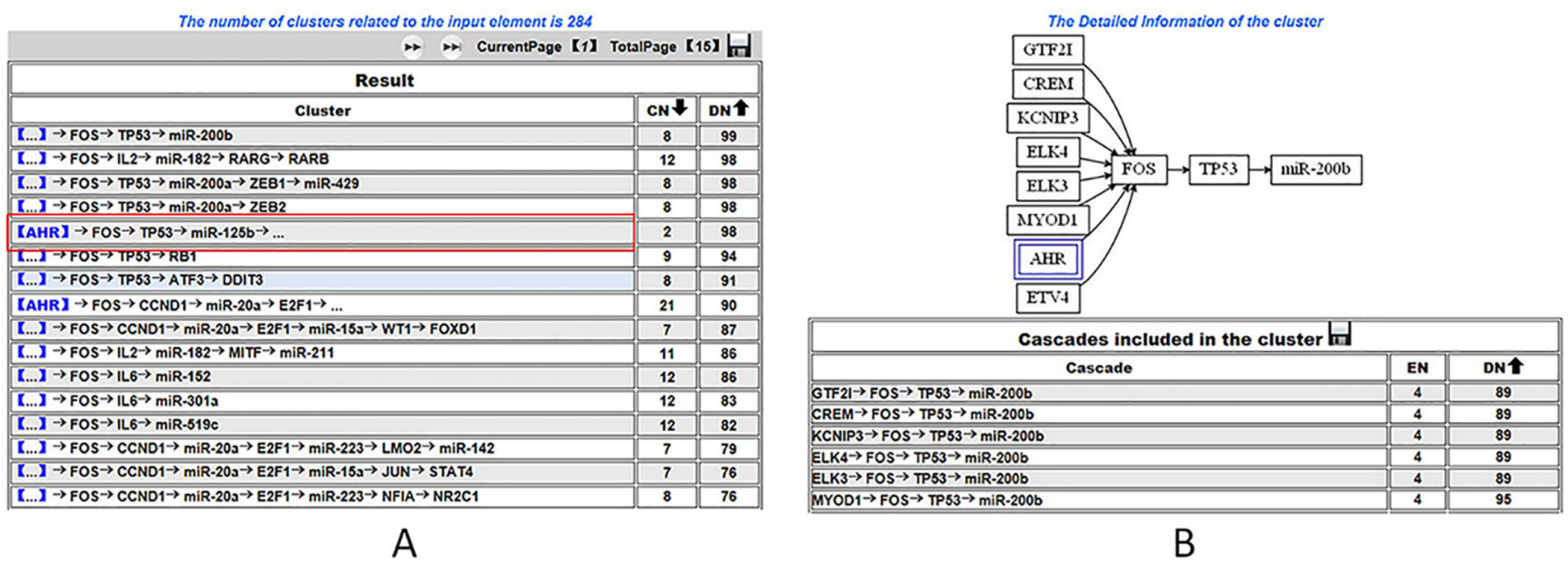
Illustration cluster search result by *AHR*.

In the cluster searching, “…” is used to represent the combined elements in different cascades. For example, we queried clusters related to *AHR* to illustrate the search procedure. After *AHR* was selected in the TF pull-down list, the Cluster button was clicked to retrieve the results. On the result page, each line presents a cluster. In each cluster, one or more cascades contain the inputted factor *AHR*. The cascade number (CN) and cluster annotated diseases number (DN) are also displayed, in which CN indicates the number of cascades included in the cluster and DN represents the total number of diseases that were annotated in each cascade of the cluster. In the listed cluster, *AHR* is displayed in blue brackets. If *AHR* is located in the “…”, then it would be marked in the same format (as shown in Fig 3A). For example, when the integrated cluster is clicked, such as in the cluster marked with the red box in Fig 3A, it is visualised as a network, in which the inputted regulator *AHR* is surrounded by blue boxes (as shown in Fig 3B). Furthermore, the cascades included in the cluster are also listed at the bottom of this page. If we click a cascade, then it executes a cascade search as described in the cascade search. In addition, when a desired disease is also selected as input, we also provide the number of factors that are related to the inputted disease (DEN) and coloured these inputs in red. Furthermore, we support the combinatorial searches. Users can input any combination of TF, miRNA or disease simultaneously. All of the query results in each step can be downloaded by clicking the save button presented on each results page.

**Fig 4:**
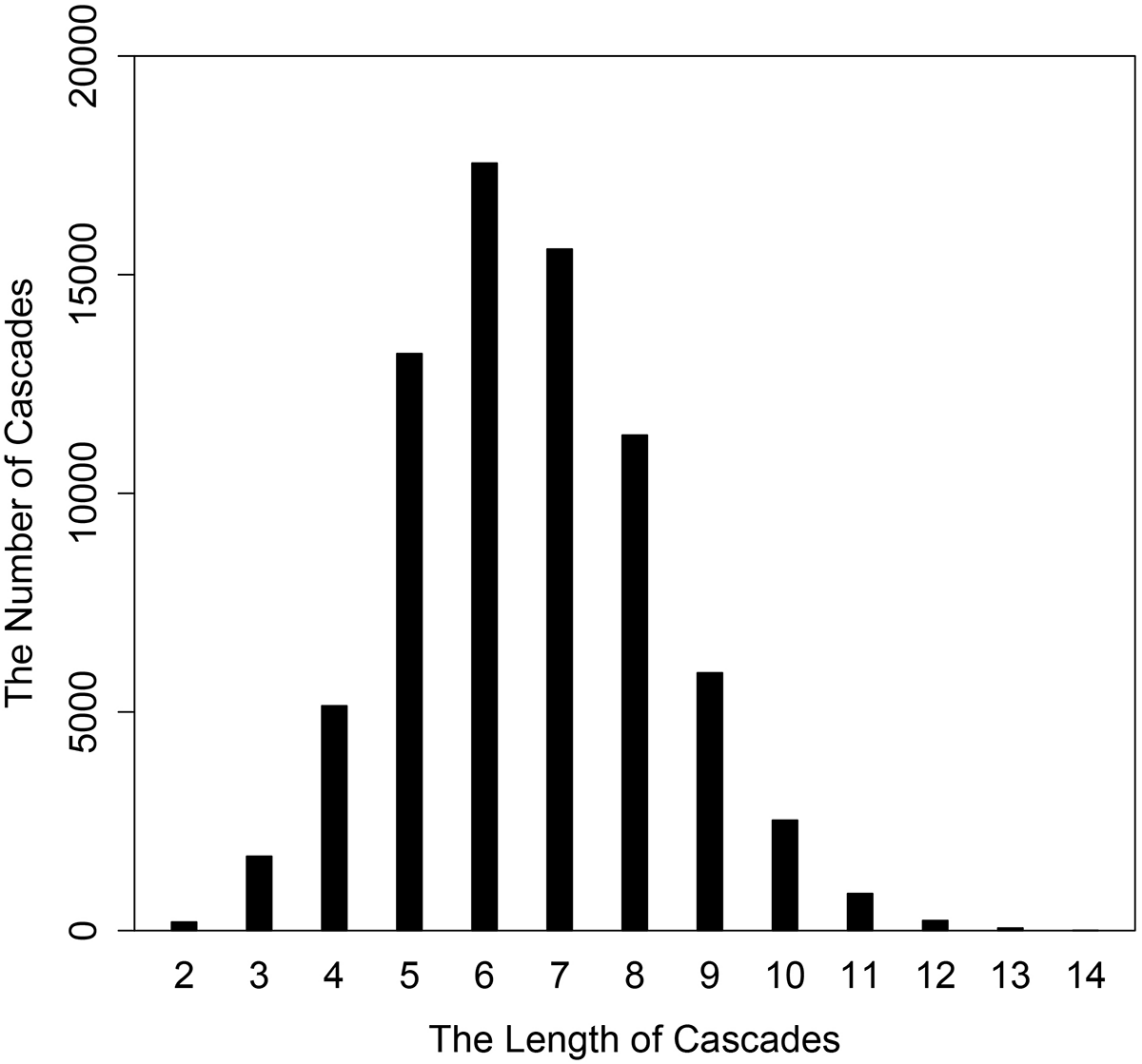
Bar plot of the length distribution of the cascades.

**Fig 5:**
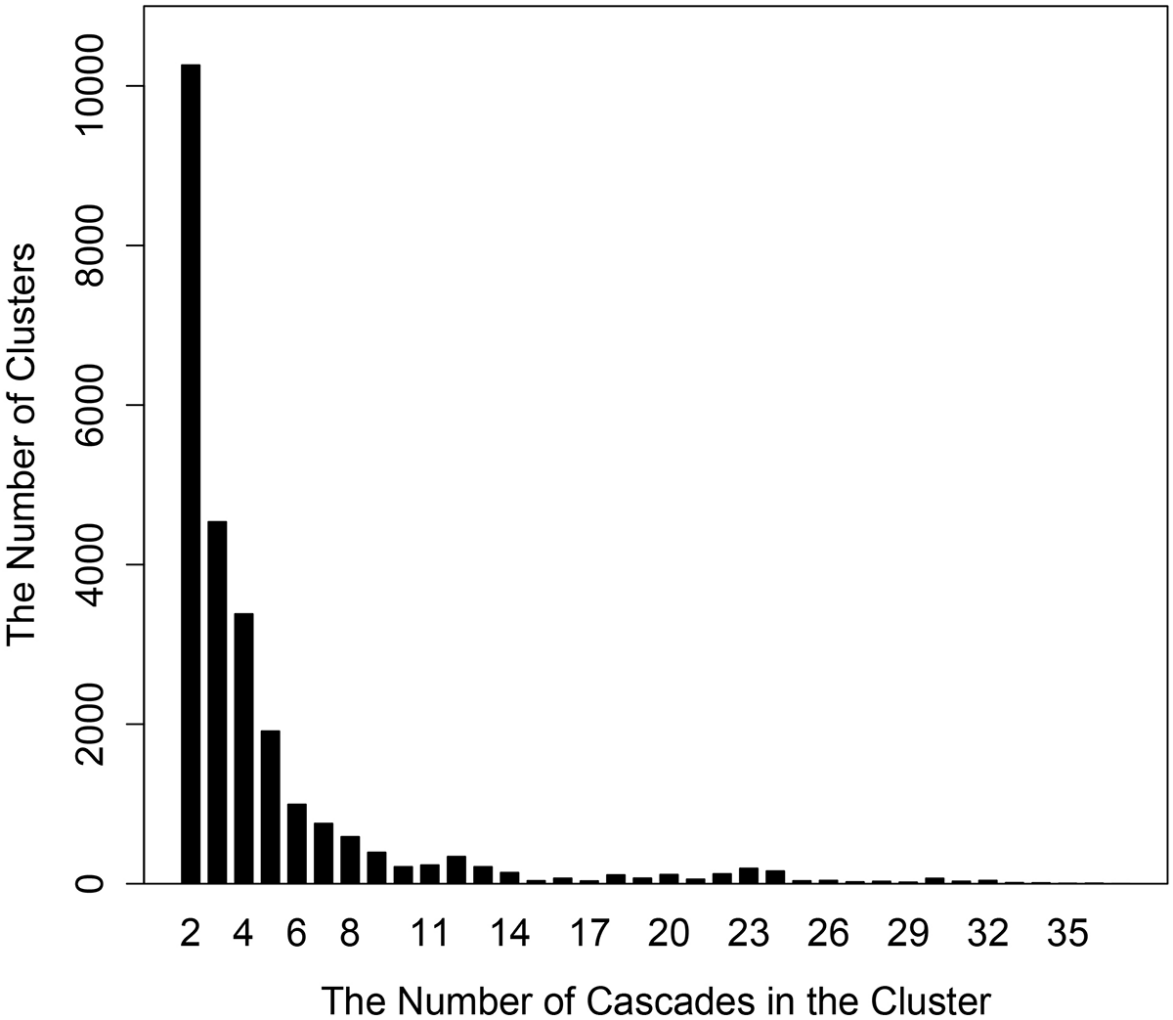
Bar plot of the number of cascades in the clusters.

We calculated the length distribution of the cascades (as shown in Fig 4). The average length of the cascades was 6.58 and the longest cascade consisted of 14 elements. We also calculated the number of cascades in the clusters (as shown in Fig 5). The average number of cascades in one cluster was 4.71 and most clusters have 2 cascades.

To globally evaluate our database to detect disease-related TFs and miRNAs, we retrieved 3673 cascades with a DAR more than 80% (S1 Table). We assumed that the elements in cascades with a DAR more than 80% were more likely to be associated with the annotated diseases. These retrieved cascades indicated novel disease associated TFs or miRNAs. When dissecting these cascades, 516 novel disease-TF pairs and 173 novel disease-miRNA pairs that were not reported before were obtained (S2 Table). All these pairs involved 21 diseases (S3 Table), 219 TFs and 85 miRNAs (S4 Table). The 21 diseases and 304 TFs/miRNAs (219 TFs and 85 miRNAs) were coupled pairwise and defined 6384 pairs as background dataset. We performed the hypergeometric test to detect the statistical significance of potential disease related TFs and miRNAs. Next, we inputted these pairs into PubMatrix [11], a database that rapidly and systematically reported back the frequency of co-occurrence between pair-wise comparisons in PubMed (S5 Table). The co-occurrence items in PubMed indicated that there existed associations between them. We presumed that the co-occurrences of diseases and TFs or miRNAs in more than 100 literatures were reliable relationships. Finally, we totally obtained 514 pairs. In these 514 pairs, there were 93 relationships also presenting in the 689 novel disease and TF/miRNA pairs (516 novel disease-TF pairs and 173 novel disease-miRNA pairs). The *p*-value of the hypergeometric test is 1.52×10^–7^, confirming that our database is reliable in detecting disease-related TFs and miRNAs. Our database is flexible, and users can adjust the DAR for reliable diseased-related TFs and miRNAs.

### Case study

TFs and miRNAs can both contribute to disease pathology. In our study, we expect that the retrieved regulated cascades would predict new disease associated genes and provide candidate therapy targets. We searched breast cancer in ^TM^REC and ordered the results by score. There was a cascade named *Src* → *miR-145* → *MYC* → *YBX1* → *EGFR* → *miR-21* → *STAT3* → *AKT1* → *miR-181c* with a high score 2.468. The DAR of the cascade about this disease was 77.8%. Almost all these elements in the cascade were associated with breast cancer except *YBX1* and *miR-181c*. Both the score and DAR indicated that this cascade was highly associated with breast cancer. Thus, we inferred that *YBX1* and *miR-181c* was also related to this disease. We resorted to the published literature to validate our predication. Dunn SE *et al* expressed *YB-1* in mammary epithelial cells and demonstrated the induction of *YB-1* promoted phenotypes associated with malignancy in three-dimensional breast acini cultures. The authors concluded that *YB-1* enabled self-renewal and the development of aggressive breast tumors [12]. Although there was no direct evidence pointing out *miR-181c* associating with breast cancer currently, several studies had demonstrated that *miR-181c* was implicated in other types cancers [13–15]. The involvement of *miR-181c* in the breast cancer was worth concern. We also inputted leukemia into the database to retrieve the leukemia association cascades. After ranking the returned results, we found that *SP1* was strongly indicated to be associated with leukemia. Zhou *et al* report a phenomenon that arsenic trioxide (ATO) treatment induced cell death in acute promyelocytic leukemia (APL) cell line HL-60 accompanied by inhibition of the human telomere reverse transcriptase (hTERT) activity, a critical enzyme responsible for the control of cell replication and transformation in cancer cells. Further study showed that transcription factors *SP1* was also suppressed by ATO strengthening ATO-induced cell growth inhibition and apoptosis. A novel mechanism of action of ATO for the treatment of APL was provided [16]. We also illustrated lymphoma for disease study. While inputting lymphoma into ^TM^REC and ranking the results, a cascade named *TP73* → *miR-145* → *MYC* → *miR-15a* → *MYB* → *miR-155* → *SPI1* → *miR-338* with the highest score was obtained. The DAR was 87.5% and *MYB* was predicted to be associated with lymphoma. Houlston RS *et al* performed a GWAS meta-analysis identifying new lymphoma susceptibility loci. Interestingly, one of the loci, *rs7745098* maps to between *HBS1L* and *MYB*. This finding indicated that *MYB* probably related to lymphoma and provided further insight into lymphoma. All these above instances demonstrated the utility of our database. ^TM^REC provides an abundant resource to identify novel disease related TFs or miRNAs and is helpful in the understanding of aetiology and therapy.

## Discussion

We developed a database named ^TM^REC, which can be used to investigate regulatory cascades among TFs and miRNAs in the context of human diseases. The disease related cascades are very helpful for the deep investigation of the underlying regulatory mechanisms of potential disease genes and prediction of novel therapeutic targets. For specific cascades, we extract the upstream and downstream relationships among different regulators and provide candidate key regulators in human diseases. Importantly, the loops formed between the miRNA and transcription factor are also essential in understanding the stable network formation in diseases or differentiation [17]. These loops will provide feedback or forward regulation and enhance the regulatory function of TFs and miRNAs. Sun *et al* systematically explored feed-forward loops (FFLs) consisting of miRNAs, transcription factors (TFs) and the effects of GBM-related genes in glioblastoma. They identified six miRNAs that might play important roles in GBM [18]. Nazarov *et al* combined time-series microarray data to analyse the interplay of miRNAs, transcription factors and target genes and derived some interesting conclusions [19]. We will investigate the regulatory loops between TFs and miRNAs in our future work. We propose that with the expansion of experimentally validated TF and miRNA regulations as well as the discovery of new disease related TFs and miRNAs, the database will become more precise and powerful. We will regularly update our database when the underlying data source expands at a satisfactory scale.

## Materials and Methods

### Data source

To retrieve more credible regulatory cascades, the relationships between TFs and miRNAs regulation employed in our database were all experimentally validated.

#### TF-miRNA data

TF regulations to miRNA were downloaded from the curated references database, TransmiR (version 1.2) [20]. TransmiR manually surveyed reports in the literature and recorded TF—miRNA regulatory relationships supported experimentally. In total, we obtained 650 relationships between 154 TFs and 175 miRNAs (S6 Table).

#### TF-TF data

We retrieved TFs interactions from TRANSFAC (version 12.4). TRANSFAC is a knowledge base database providing published data on eukaryotic transcription factors and their experimentally-proven regulated genes. In this study, we only examined the regulatory cascades of regulators, and we only retained the interactions between TFs. The total number of literature-supported interactions between TFs was 671 observed (S7 Table).

#### MiRNA-TF data

Experimental validated miRNA regulations were acquired from the databases of TarBase 5.0 [21] and miRecords [22]. To obtain a sufficiently sufficient high confident miRNAs and their regulated TFs, we obtained the union of the two miRNA target databases and obtained 288 pairs between 131 miRNAs and 136 TFS (S8 Table).

#### Disease information

The miRNA disease information was obtained from miR2Disease [23] and Human miRNA & Disease Database (HMDD) [24]. In addition, we marked each TF with the associated diseases by the records in GeneCards database (S9 Table).

**Fig 6:**
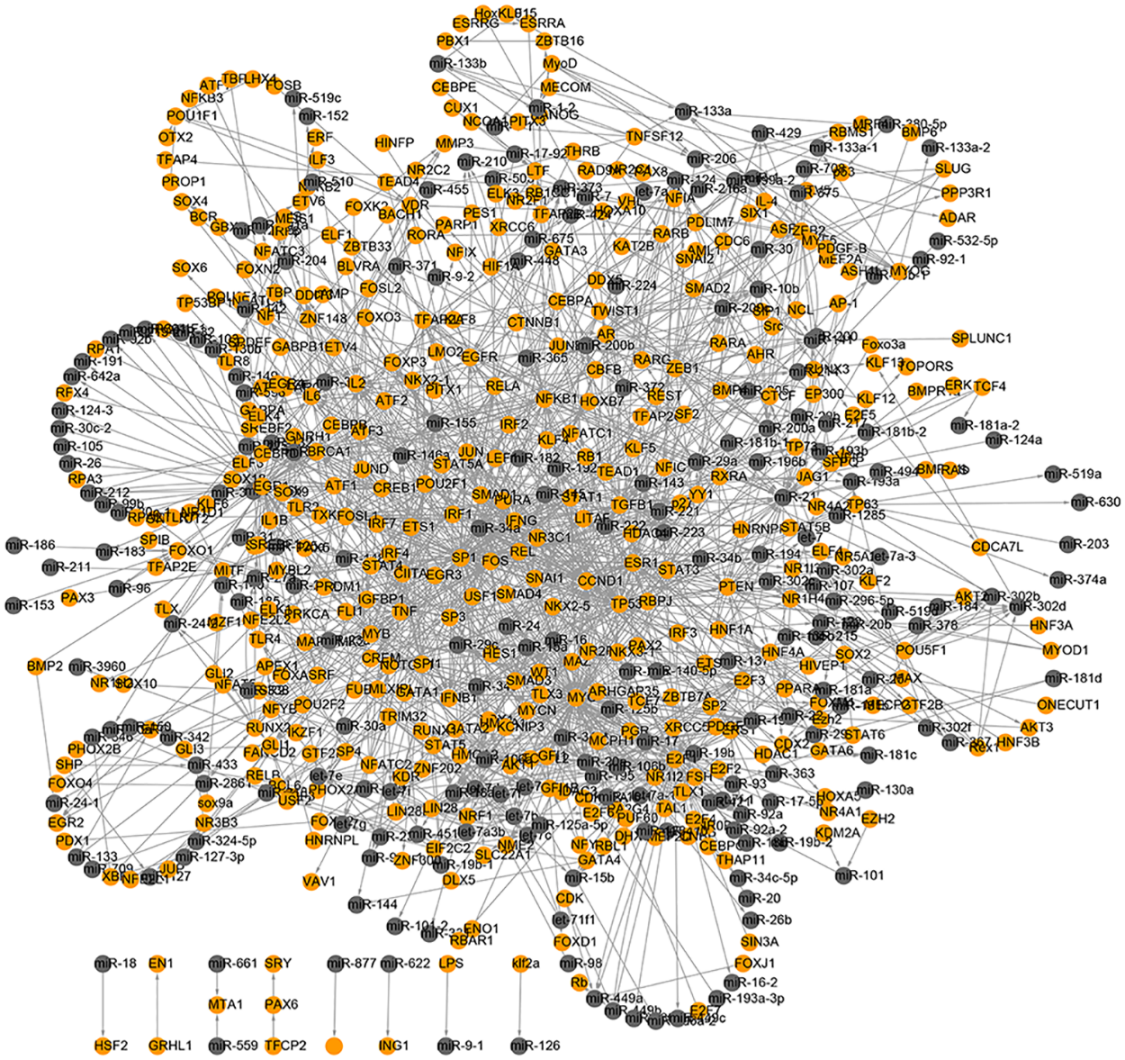
Human TF and miRNA regulatory network. Yellow circles represent TFs and gray ones represent miRNAs.

When connecting all these regulating relationships from corresponding databases, we obtained a regulatory network. The nodes represent TFs or miRNAs recorded in these databases and the edges represent the regulating relationships between these TFs and miRNAs. In order to get a global view of these interactions, we use the cytoscape software to graphically visualize the network (Fig 6). Cytoscape is an open source software platform for visualizing and analyzing complex networks, with molecular species represented as nodes and intermolecular interactions represented as links, that is, edges, between nodes [25]. After importing the regulating pairs between TFs and miRNAs into cytoscape, the software displayed these interactions as a two-dimensional network. Finally, 412 TFs and 226 miRNAs were identified in the entire network. The largest component of the TF-miRNA network includes 1610 edges, and it contains 99.4% of the elements in the network.

Next, the BFS traversal method which was used in our previous study [8] was employed to search cascades in this network. The BFS algorithm is one of the most commonly used graph algorithms and systematically expands all elements to check the graph. In this study, the regulatory network can be treated as a directed graph. We identified all paths between every two elements and defined these directed acyclic paths with more than 2 elements as cascades. The procedure of identifying cascades from a network is illustrated in S1 File. Furthermore, if a cascade completely contained in another cascade, it would be filtered (Details in S1 File). In addition, we integrated disease information into the filtered cascades and computed the disease annotation ratio (DAR). The DAR was calculated as the ratio of the number of elements related to a specific disease in one cascade and the total number of elements of the cascade. A higher DAR received by a cascade indicated a closer relationship with the disease. If the DAR reached 100%, all elements in the cascade have been reported to be related to the disease and the cascade was predicted to contribute to the pathology. With regard to the prediction of disease related TFs or miRNAs, the unassociated elements in the cascade with a high DAR were more likely to have relationships with a certain disease. We further proposed a score to evaluate the results. A basic assumption in our study is that the upstream elements are more important in the explanation of aetiology. Thus, we scored the cascades by the location of the disease related elements. The score was calculated as

$$score\;=\;\sum_{i\;=\;1}^{n}1/location\left(n_{i}\right)$$

where *n*_*i*_ represented the disease related element and *location*(*n*_*i*_) represented the location of the node in the cascade. A high score indicated the disease related elements mostly located in the upstream of the cascade and marked the importance of the cascade in the disease study. Combine the score with DAR, we can effectively identify interesting cascades.

**Fig 7:**
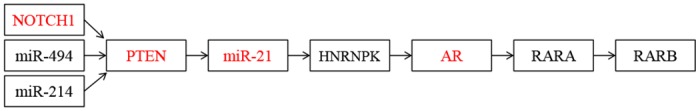
Illustration of clustering of three cascades. The three cascades are almost the same except the beginning elements. We clustered them to provide comparable analysis for detecting disease related cascades.

In the results of cascades filtration, we found that some cascades were nearly the same except the beginning element or the ending element. These similar cascades may relate to the same disease but differ in the initial or ending cascade. For example, there were three cascades related to breast cancer: *NOTCH1*→*PTEN*→*miR-21*→*HNRNPK*→*AR*→*RARA*→*RARB*; *miR-494*→*PTEN*→*miR-21*→*HNRNPK*→*AR*→*RARA*→*RARB*; *miR-214*→*PTEN*→*miR-21*→*HNRNPK*→*AR*→*RARA*→*RARB*;

All these three cascades almost the same except the beginning element. The different initial of the cascade may lead to different results and contribute distinguishingly to related diseases. In order to provide comparable analysis between these cascades, we integrated the cascades into clusters. The cascades united into a cluster should satisfy following restriction. First, the number of the elements in these cascades is the same. Second, the different factors only exist in the beginning or the end of the cascades (Details in S1 File). We provided cluster visualization in the Cluster search in the database. The above three cascades were integrated as shown in Fig 7. It intuitionally manifested the relationships between these cascades and provided comparable results for dissecting disease aetiology. Finally, ^TM^REC is developed using JSP, tomcat 6.0 and MySQL5.0 and runs under Cent OS 5.5 system.

## Supporting Information

S1 File The procedure of BFS algorithm and clusters identification. (DOC)

S1 Table The 3673 cascades with DAR more than 80%. (XLS)

S2 Table The 689 novel disease and TFs or disease and miRNAs pairs. (XLS)

S3 Table The 21 diseases involved in the novel disease and TFs or disease and miRNAs pairs. (XLS)

S4 Table The 304 TFs/miRNAs involved in the novel disease and TF/smiRNAs pairs. (XLS)

S5 Table The results of searching in PubMatrix. (XLS)

S6 Table The 650 TF-miRNA pairs between 155 TFs and 175 miRNAs. (XLS)

S7 Table The 671 TF-TF pairs. (XLS)

S8 Table The 288 miRNA-TF pairs between 131 miRNAs and 136 TFs. (XLS)

S9 Table The disease information about TFs and miRNAs. (XLS)
