# Supplementary material for: TMREC: A Database of Transcription Factor and MiRNA Regulatory Cascades in Human Diseases
Source: PLoS One. 2015 May 1;10(5):e0125222. doi: 10.1371/journal.pone.0125222 (PMC4416930; doi:10.1371/journal.pone.0125222)
Supplement: S1 File — (DOC) [file pone.0125222.s001.doc]

# The procedure of BFS


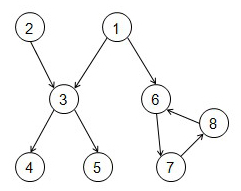


Figure 1. An example of how to find the pathways

For each node in the graph, we execute the same procedure as follows. Let’s take node 1 as an example.

(1) Select node 1 as the root node and mark all other node as unvisited nodes. Inspect all of the child nodes of node 1 (node 3 and node 6), and store the parent-child relationships of these nodes in a queue. Then mark these nodes as visited;

(2) For each of the child nodes (node 3 and node 6), in turn, inspect their child nodes that are unvisited and also store the parent-child relationships of these nodes. These nodes are also marked as visited;

(3) Repeat step 2, until there are no more unvisited nodes that can be reached by node 1. For example, the child node of node 3 is node 4 and node 5, the child node of node 6 is node 7 and the child node of node 7 is node 8;

(4) For each of the leaf node, back up to find the parent nodes in turn. Thus, we can identify all of the directed acyclic paths between node 1 and all other nodes. The directed acyclic paths with more than 2 nodes were considered to be a cascade. After applying the procedure to each node of the graph, we got all the cascades about the Figure 1. We listed these cascades in Table 1.

Table 1. All the cascades identified in Figure 2 related to each node by BFS.

| **The node selected as root** | **The cascades identified by BFS** |
| --- | --- |
| 1 | 1🡒3🡒4; 1🡒3🡒5; 1🡒3;1🡒6🡒7🡒8; 1🡒6🡒7; 1🡒6; |
| 2 | 2🡒3🡒4; 2🡒3🡒5; 2🡒3 |
| 3 | 3🡒4; 3🡒5; |
| 4 |  |
| 5 |  |
| 6 | 6🡒7🡒8; 6🡒7 |
| 7 | 7🡒8🡒6; 7🡒8 |
| 8 | 8🡒6🡒7; 8🡒6 |

Finally, there is a filtration in the results of BFS. For example, the cascade 3🡒4 is retrieved when the node 3 starts as the root It is completed contained in the cascade 1🡒3🡒4. So, it is merged into the cascade 1🡒3🡒4. The filtered cascades are listed in Table 2.

Table 2. All the cascades after filtration.

| **The node selected as root** | **The cascades after filtration** |
| --- | --- |
| 1 | 1🡒3🡒4; 1🡒3🡒5; 1🡒6🡒7🡒8; |
| 2 | 2🡒3🡒4; 2🡒3🡒5; |
| 3 |  |
| 4 |  |
| 5 |  |
| 6 |  |
| 7 | 7🡒8🡒6; |
| 8 | 8🡒6🡒7; |

1. **The procedure of identifying clusters**

In the result of cascades filtration described above, we found that some cascades were almost the same except the begin node or the end node. For example, the cascade 1🡒3🡒4 and 2🡒3🡒4 are almost the same except the beginning node 1 and node 2. In order to make a global view of the cascades, we integrated the cascades into clusters. The cascades united into a cluster should satisfy the following restriction. First, the numbers of the elements in these cascades are same. Second, the different factors only exist in the beginning or the end of the cascades. All the clusters are listed in Table 3.

Table 3. All the clusters after cascades integration.

| **The clusters after cascades integration** | **The cascades in the cluster** |
| --- | --- |
| …🡒3🡒4 | 1🡒3🡒4; 2🡒3🡒4; |
| …🡒3🡒5 | 1🡒3🡒5; 2🡒3🡒5; |
| 1🡒3🡒… | 1🡒3🡒4; 1🡒3🡒5; |
| 2🡒3🡒… | 2🡒3🡒4; 2🡒3🡒5; |

“…” represents the combined elements in different cascades.
